# Exploring Consequences of Predator Stress on Behaviors of Mice Lacking Trace Amine-Associated Receptor 5 (TAAR5)

**DOI:** 10.3390/cells15010039

**Published:** 2025-12-25

**Authors:** Vsevolod V. Nemets, Vladimir P. Grinevich, Evgeniia N. Petrunina, Evgeny A. Budygin, Raul R. Gainetdinov

**Affiliations:** 1 Institute of Translational Biomedicine, Saint Petersburg State University, 199034 Saint Petersburg, Russia; v.v.nemets@spbu.ru (V.V.N.); st086960@student.spbu.ru (E.N.P.); 2 Department of Neuroscience, Sirius University of Science and Technology, 354340 Sirius, Russia; grinevich.vp@talantiuspeh.ru; 3 Department of Gerontology, Wake Forest University School of Medicine, Winston-Salem, NC 27157, USA; ebudygin@wakehealth.edu

**Keywords:** TAAR5, dopamine, predator stress, voltammetry, trace amine-associated receptor, anxiety, depression

## Abstract

**Highlights:**

**What are the main findings?**

- In mice lacking TAAR5, enhanced accumbal DA release, reduced anxiety, and an increased hedonic state were demonstrated.
- TAAR5-KO mice were active in coping with predator exposure but were vulnerable to the short-term stress consequences, showing robust anxiety and lowered exploration.

**What are the implications of the main finding**

- Observed behavioral distortions due to TAAR5 deletion imply further pharmacological exploration into the receptor as a drug target for reward deficits, anhedonia, and stress.
- A robust increase in post-stress anxiety of TAAR5-deficient mice suggests alteration of recovery from stress by potential receptor antagonists.

**Abstract:**

Recent studies indicated a connection between trace amine-associated receptor 5 (TAAR5) and emotional behaviors related to anxiety and depression; however, the neurobiological basis of this link is still unclear. Using mutant TAAR5 knockout (TAAR5-KO) mice, we explored the consequences of receptor deletion on dopamine (DA) dynamics in the ventral striatum and stress-related behaviors. Voltammetric measurements of DA in the nucleus accumbens (NAc) coupled with electrical stimulation of the ventral tegmental area (VTA) revealed that mice lacking TAAR5 display a greater DA release, while its reuptake is not affected. Behaviorally, mutants were significantly less anxious in the elevated plus maze (EPM) and consumed more sucrose in comparison with wild-type (WT) controls. The new object recognition test (NOR) did not uncover a difference between these genotypes. During predator (rat) stress exposure, mutant and WT mice showed quite distinct responses versus the behavior observed in stressless conditions. Control animals demonstrated a substantial increase in “freezing” (a sign of passive coping), while “running” and “exploring” patterns (signs of active coping) were significantly extended in mice lacking TAAR5. Short-term consequences of stress were explored 24 h following the predator exposure. The absence of TAAR5 did not prevent or reduce stress-induced anxiety in the EPM. In fact, the level of anxiety in mutants reached that observed in control mice. Furthermore, activity in NOR was significantly decreased in mice lacking TAAR5 but not in WT animals. On the other hand, predator exposure resulted in impaired NOR in the WT control, whereas mutants’ performance was not altered. These findings indicate that TAAR5 deletion leads to significant DA imbalance, which might at least partly explain the better stress-coping strategy and other stress-induced behavioral consequences observed in mutant animals.

## 1. Introduction

Stress is capable of inducing substantial changes in the functioning of the neurohumoral system that may often trigger or escalate serious pathological conditions such as anxiety. The use of monoamine reuptake inhibitors and cognitive behavioral therapy are a long-established approaches to managing symptoms of this disease. Recent advancement in the treatment of this emotional disorder is evident in cognitive behavioral therapy. Indeed, so-called digital therapeutics, which are delivered via evidence-based software products, have become beneficial tools to reduce symptoms of generalized anxiety disorder over time. There are also some noticeable improvements regarding the efficacy and safety of traditional medications, including serotonin and norepinephrine reuptake inhibitors. However, the discovery of principally novel pharmacotherapies is undoubtedly delayed, perhaps due to an incomplete understanding of neurobiological mechanisms of anxiety and, therefore, uncertainty of potential targets for pharmacology.

The emergent role of the TAAR5 in anxiety-related disorders is becoming a new promising direction of research [1,2,3]. TAAR5 belongs to subfamily II of trace amine-associated receptors (TAAR), which are sensory G protein-coupled receptors that detect biogenic amines, products of decarboxylation of amino acids. TAAR5 can be activated by tertiary amines, bacterial by-products in the gut, particularly by trimethylamine [4]. TAAR5 is the most highly conserved TAAR subtype among all characterized mammalian species investigated so far. This member of the TAAR5 family was found in different limbic brain areas, which are primarily involved in anxiety regulation and stress response, including the nucleus accumbens, hippocampus, hypothalamic nuclei, and amygdala [1]. Genetic deletion of TAAR5 in mice results in the anxiolytic- and antidepressant-like phenotypes [1]. Perhaps, this receptor can be targeted for developing new, more effective medications for the treatment of anxiety and depression disorders.

Since trace amines are known to modulate monoamine neurotransmission, it was assumed that TAAR5 might be involved in the regulation of DA [5]. The pharmacological finding that the putative TAAR5 agonist enhances the turnover rate of dopamine (DOPAC/DA ratio) measured in striatal tissue [6] agrees with this assumption. Moreover, mice lacking TAAR5 display an increase in the number of dopaminergic neurons along with increased DA and its metabolites tissue content as well as augmentation of related signaling events [2,4,7], which suggest enhanced DA release. However, direct evidence that DA release is indeed altered in these mice has been missing.

Therefore, the first goal of this study was to explore whether DA release is modified in the absence of the TAAR5 functioning in the mouse brain. Fast-scan cyclic voltammetry (FSCV) recordings coupled with electrical stimulation of DA cell bodies in the ventral tegmentum area (VTA) are one of a few approaches comprehensively used to evaluate functional release and consequent uptake of endogenous DA in the rodent brain in vivo [8]. Using this technique, we tested the premise that the stimulation of VTA will result in enhanced DA release in the NAc of the TAAR5-KO mice.

Previous behavioral studies indicated a reduction in anxiety-like behaviors in TAAR5-KO mice compared to wild-type controls [1]. Based on this unique phenotype, we hypothesized that genetic removal of the TAAR5 might result in the development of lower stress susceptibility.

There are a variety of laboratory models, which are recommended for the exploration of stress effects in rodents [9]. The exposure to a predator (predator stress) or to an aggressive opponent (social defeat) is most efficient and therefore commonly used models [9,10,11,12]. By comparing these stress models on mice, it was revealed that a higher level of behavior despair was elicited by predator stress, while social defeat resulted in more robust social avoidance [13]. Furthermore, the predator stress elicited phenotype in mice reflects important aspects of depression and generalized anxiety disorders [13]. Therefore, the second goal of this study was to explore the consequences of predator stress exposure on the anxiety level and cognitive ability of TAAR5-KO mice.

## 2. Materials and Methods

### 2.1. Animals

Male 3–5 months old WT and TAAR5-KO mice [1,2,4,14,15], and male Dark Agouti rats (300–350 g) were used in our study. Mice and rats housed in their standard plastic cages (five mice or one rat per cage) were maintained on a 12 h light/12 h dark cycle with food and water available ad libitum. All procedures involving animals were conducted in accordance with the Guide for the Care and Use of Laboratory Animals: Eighth Edition [16] and the animal study protocol was approved by the Institutional Animal Care and Use Committee at St Petersburg University (protocol № 131-03-3, 28 February 2025).

### 2.2. Voltammetric Measurements of DA in the NAc

Electrically evoked DA release was recorded by FSCV in the NAc of anesthetized TAAR5-KO and WT mice using the same protocols we utilized for rat studies [17,18] but with some species-specific adjustments. Mice were anesthetized by isoflurane (1% isoflurane/1 L/min O_2_). Holes were drilled in the scalp in order to implant electrodes into the brain. A stimulating electrode was inserted into the VTA (AP: −3.0 mm, ML: 0.5 mm, DV: above 4.5 mm); a carbon fiber working microelectrode (exposed fiber length 75–100 μm; diameter 6 μm) was placed into the NAc (AP: 1.2 mm, ML: 0.8 mm, DV: above 4 mm) and an Ag/AgCl reference electrode was implanted into the brain tissue of the contralateral hemisphere. The electrodes were connected through the head stage to a computer running the specialized software. Extracellular DA was detected due to its oxidation current at the carbon fiber electrode every 100 ms by applying a triangular waveform (−0.4 V to +1.3 V and back to −0.4 V vs. Ag/AgCl, 400 V/s). The DA signal was verified by a background-subtracted cyclic voltammogram characterized by oxidation and reduction peaks occurring at +0.6 and −0.2 V, respectively [17,19,20,21,22]. For pharmacological verification of working electrode placement in the NAc, raclopride (a D2 receptor antagonist) and GBR 12909 (a DA transporter inhibitor) were used [19,23]. DA uptake was assessed by applying Michaelis–Menten kinetics to determine maximal uptake rate (Vmax) and Km value [8,23,24]. All uptake data were analyzed using a specialized online program [25].

### 2.3. Predator Exposure Procedure

For predator exposure (stress) studies, we used the resident-intruder paradigm, which is effective in achieving stress in intruder animals as has been shown in our previous studies [17,18]. To achieve a stressful condition, the WT or TAAR5-KO mouse (intruder) was exposed once to the resident Dark Agouty rat (predator) in the rat host cage (27.5 × 16 × 15 cm, length × width × height). The latter is known to exhibit high levels of anxiety and aggressiveness [26]. On the other hand, the TAAR5-KO genotype is known as an olfactory cue-deficient so being not able to efficiently identify the predator. Consequently, this experiment was designed to minimize the possibility of losing and wounding mutant mice. Thus, the procedure was performed in two 5 min phases: the first one was a direct mouse–rat interaction, the second was a protected interaction, while the mouse was separated from the rat by a protective wire cylinder. During the direct interaction phase, in case of signs of violent aggression (bites) from the rat, the procedure was interrupted, and the mouse was protected by a wire cylinder for the remaining time. Such a combination of the direct mouse–rat interaction with the follow-up protected stress-prolongation phase provided with sufficiently high level of stress exposure for the purposes of our study. To assess the effects of the predator stress per se for both TAAR5-KO and WT mice, we used control mice of each genotype, which underwent stressless interaction with a still object mimicking a rat in size (it was conducted in an identical cage to the predator cage). All encounters of the procedure were video recorded and ethologically analyzed using blinded numeration. Detailed quantitative analysis was performed using a special program for semi-automatic calculation of behavioral elements. Observed behaviors of the intruder mice during the direct physical contact phase were enumerated (as a duration of time). All behavioral studies were conducted within 1:00 p.m.–5:00 p.m. in order to minimize the effects of the circadian rhythm [27].

For the analysis of stress-related and coping-adaptive responses during the predator exposure or stressless condition, we quantified the following ethological elements of stress-related behaviors: grooming (self-directed cleaning movements of the head or body), freezing (complete body immobility), exploring (sniffing—active investigation of the environment or objects through rhythmic nose and vibrissae movements), climbing and rearing (vertical exploratory movements) and other forms of behaviors such as standing and walking. Also, we identified behavioral patterns, which represent active (running) or passive (freezing) coping with stress due to the predator exposure [13,17,18].

Following the single predator stress procedure, all animals were housed individually for a 24 h period. After that, animals underwent a standard behavioral test battery to evaluate depression-like behaviors.

### 2.4. Stress Follow-Up Behavioral Studies

To evaluate baseline anxiety and the impact of single predator stress, the elevated plus maze (EPM) test was performed [28,29]. Individually housed TAAR5-KO and WT mice were habituated to the testing room before being placed in the center of the maze, which consisted of two open arms (30 × 5 cm) perpendicular to two closed arms (30 × 5 cm) at a height of 50 cm above the ground. Spontaneous animal’s exploration of the open and closed arms was recorded for 300 s using a video-tracking system (EthoVision XT 11.5, Noldus, Noldus Information Technology, Wageningen, The Netherlands). Time spent by the mouse in each pair of open or closed arms was taken into account. After each trial, the apparatus was cleaned with a 3% hydrogen peroxide solution to eliminate residual olfactory cues.

To evaluate cognitive deficits associated with predator stress, the NOR test was performed [30,31,32,33]. This test was selected due to its established efficacy in assessing the cognitive consequences of a single social defeat stress in our previous studies [17,18]. To minimize additional stress, all NOR procedures were conducted in the animals’ individual home cages [34,35].

The sucrose preference was measured in TAAR5-KO and WT mice using a two-bottle choice paradigm [13,17]. Bottles with water and 6% sucrose solution were placed in individual cages 3 days before (sucrose habituation phase) and 24 h after the predator stress (test phase). No food or water deprivation was applied before the test. The consumption of water and 6% sucrose was assessed for 24 h. Sucrose preference was calculated as a ratio of the amount of 6% sucrose solution to the total amount of consumed fluids and was expressed as a percentage [36,37].

### 2.5. Statistical Analysis

The voltammetrically measured DA oxidation current (nA) was calibrated and converted into a molar concentration of released DA (µM). The D’Agostino-Pearson omnibus normality test was used to evaluate whether the values followed a Gaussian distribution, and then, in case of normal distribution, we used a parametric unpaired two-tailed *t*-test; otherwise, the nonparametric Mann-Whitney U-test was utilized. For the comparison between two groups (WT and TAAR5-KO), a Two-Way ANOVA (analysis of variance) was utilized. Sidak’s or Dunn’s multiple comparison test was used to distinguish prominent differences. All analyses were carried out using GraphPad Prism (version 8.0.1, San Diego, CA, USA). The data were expressed as a mean ± SEM with a criterion for significance set at *p* ≤ 0.05.

## 3. Results

### 3.1. Effect of TAAR5 Deletion on Dynamics of Electrically Evoked DA

**Figure 1:**
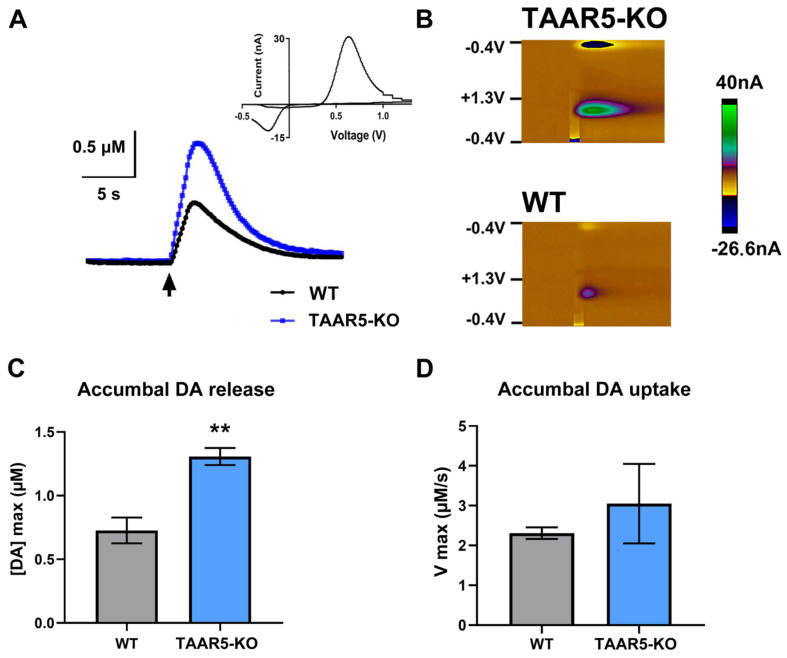
Electrically evoked accumbal DA responses in TAAR5-KO and WT mice. (**A**) Representative signaling and voltammogram of DA in the NAc in response to the VTA electrical stimulation (60 Hz, 60 pulses, 330 μA) in TAAR-KO and WT mice. Black arrow—origin point of electrical stimulation. (**B**) Representative color plots. (**C**) Accumbal DA release is expressed as a maximal peak concentration (μM). (**D**) DA uptake is expressed as a velocity (μM/s). Data are presented as mean ± SEM; ** *p* < 0.01, Mann–Whitney U-test.

FSCV recordings of DA in the NAc coupled with electrical stimulation (60 Hz, 60 pulses) of the VTA were used to explore if a deletion of TAAR5 in mice causes alterations in their DA release and reuptake. Figure 1 shows representative traces of extracellular DA in TAAR5-KO and WT mice, along with the voltammogram (Panel A) and color plot of DA (Panel B) observed in our study. The maximal amplitude of the DA signal (Panel C) was markedly greater in TAAR5-KO mice compared to WT counterparts (1.31 ± 0.07 µM and 0.73 ± 0.10 µM, respectively; *p* < 0.01).

Moreover, maximal accumbal DA uptake rate, V max, was not statistically different (Panel D) between TAAR5-KO and WT groups (3.0 ± 1.0 and 2.3 ± 0.2 µM/s, respectively). This indicates that the uptake rate is preserved in mutants.

### 3.2. Behavioral Profile Associated with Lack of TAAR5

The assessment of behavior of TAAR5-KO mice versus WT counterparts in the EPM test revealed that TAAR5-KO mice spend significantly less time versus WT mice in closed arms (104 ± 34 s vs. 190 ± 25 s; *p* < 0.05, Two-Way ANOVA with Sidak’s multiple comparison test) and more time than WT mice in open arms (249 ± 25 s vs. 158 ± 23 s, *p* < 0.05, Two-Way ANOVA with Sidak’s multiple comparison test). These data clearly indicate the difference in the basal level of anxiety between these two genotypes. At the same time, no behavioral deviation was found in the NOR test. Thus, TAAR5-KO and WT mice demonstrated fairly similar total exploration time (31 ± 6 s and 37 ± 12 s, respectively; *p* = 0.67, unpaired two-tailed *t*-test) and so in regard to discrimination index (0.3 ± 0.1 and 0.5 ± 0.1, respectively; *p* = 0.07, unpaired two-tailed *t*-test). However, there was a significant difference between these genotypes in sucrose consumption. Importantly, intake of the 6% sucrose solution was higher in TAAR5-KO mice in comparison to WT control (50.4 ± 5.9 g and 40.6 ± 2.0 g, respectively; *p* < 0.05, Two-Way ANOVA with Sidak’s multiple comparison test). Both animal groups consumed significantly more sucrose than water: the sucrose/water discrimination index was 94.3 ± 1.0% for TAAR5-KO and 93.7 ± 0.6% for WT mice. No difference in water consumption was observed between mutants and WT mice (2.7 ± 0.4 g and 2.7 ± 0.3 g, respectively).

**Figure 2:**
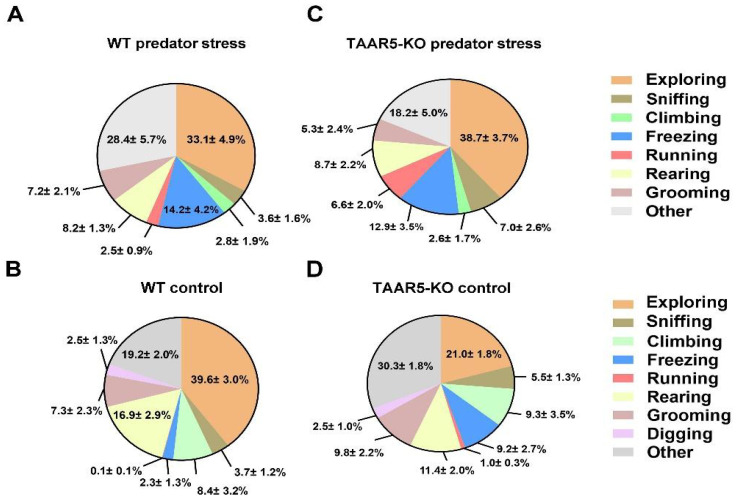
Behavioral patterns observed in TAAR5-KO and WT mice during predator stress exposure and the stressless (control) condition (**A**,**C**) demonstrate behavioral patterns in mice assessed under the predator stress; and (**B**,**D**) show behaviors under the stressless condition (interaction with the still object that mimics rat in size) in TAAR5-KO (*n* = 8) and WT (*n* = 9) mice.

**Figure 3:**
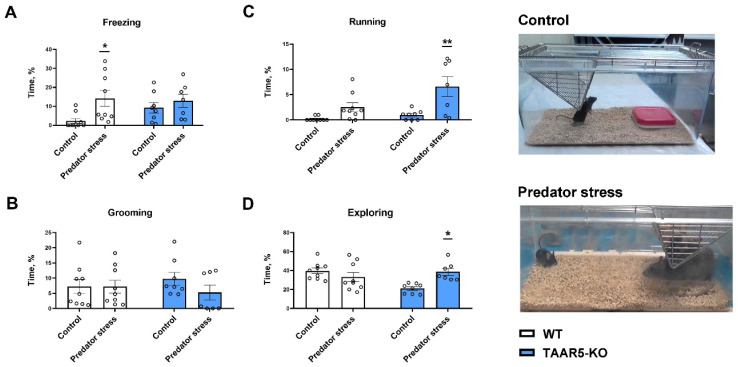
Behavioral patterns displayed by TAAR5-KO (filled columns) and WT (not filled columns) mice under the stressless (control) condition and during predator stress were denoted as (**A**) freezing, (**B**) grooming, (**C**) running, and (**D**) exploring behavior. Two-way ANOVA with Dunn’s multiple comparison test was used for the data analysis. All data are expressed as a percentage of the total time of mice interaction with the predator (mice-rat) and presented as mean ± SEM values for TAAR5-KO (*n* = 8) and WT (*n* = 9). Significance level * *p* < 0.05, ** *p* < 0.01 vs. corresponding control.

Two groups of mice behaved quite similarly in the stressless condition, which served as the control for the predator stress assessment (Figure 2B,D and Figure 3). The difference between TAAR5-KO and WT mice was found only in the exploring pattern of activity (21.0 ± 1.8% and 39.6 ± 3%, respectively; *p* < 0.01, Two-Way ANOVA with Tukey’s multiple comparison test). No significant differences between genotypes were found in “freezing” (*p* = 0.53) and other behavioral patterns.

### 3.3. Behavioral Alterations Observed During the Predator Stress Exposure

During the free interaction phase of the predator exposure procedure (Figure 2A,C), all intruder mice were equally subjected to robust aggressive attacks from resident rats.

However, behavioral responses to the threat and dangerous environment were markedly distinct between genotypes. Particularly, the behavior of TAAR5-KO mice was shifted to more active patterns, such as running and exploring, while the behavior of WT animals was shifted to a more passive form of behavior, such as freezing (Figure 2 and Figure 3).

Two-Way ANOVA with Dunn’s multiple comparison test (Figure 3) revealed that mutants displayed an increase in “running” (6.6 ± 2.0% during the stress vs. 1.0 ± 0.3% under stressless condition (control), *p* < 0,05) and in “exploring” (38.7 ± 3.7% during the stress vs. 21.0 ± 1.8% of control, *p* < 0.05). No significant alterations in these patterns were evident in WT mice (for “running” 2.7 ± 0.8% during the stress vs. 1.0 ± 0% control, *p* > 0.05; for “exploring” 33.1 ± 4.9% during the stress vs. 39.6 ± 3.0% of control, *p* > 0.05). However, WT mice preferentially demonstrated escalated “freezing” (14.2 ± 4.2% during stress vs. 2.3 ± 1.3% of control, *p* < 0.05), whereas this parameter in TAAR5-KO mice was unchanged (12.9 ± 3.5% during stress vs. 9.2 ± 2.7% of control, *p* > 0,05). “Grooming” was not significantly altered in either genotype (for mutants 5.3 ± 2.4% during stress vs. 9.8 ± 2.2% of control, *p* > 0,05; for WT mice 7.2 ± 2.1% during stress vs. 7.3 ± 2.3% of control, *p* > 0.05).

### 3.4. Behavioral Consequences Observed 24 h Following Stress Exposure

**Figure 4:**
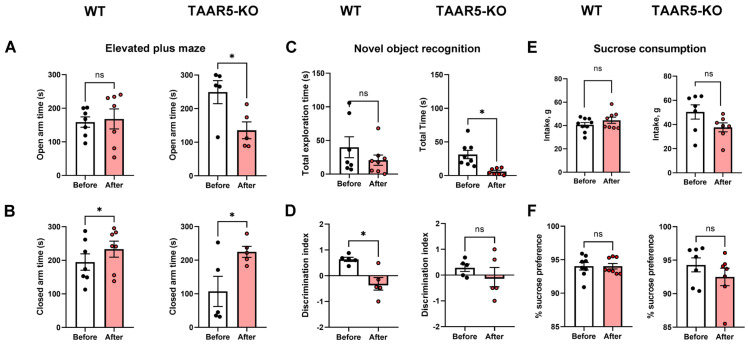
Behaviors of TAAR5-KO and WT mice before and 24 h after the single predator stress. The right columns represent behaviors of TAAR5-KO mice, and the left columns—WT. Anxiety level of TAAR5-KO and WT mice is shown as time spent in open (**A**) and closed (**B**) arms of the elevated plus maze (EPM). Novel object recognition parameters are displayed as total exploration time (**C**) and discrimination index (**D**). (**E**,**F**) show sucrose intake and preference in TAAR5-KO and WT. Statistical analysis was assessed using a paired two-tailed *t*-test. All data are mean ± SEM. * *p* < 0.05. Abbreviation ns denotes not significant. Sucrose preference (**E**) was counted as a ratio (%) of the consumed 6% sucrose solution (g) to the total fluid intake (sucrose + water, g).

The predator stress altered the anxiety-related behavior observed in the EPM test for both TAAR5-KO and WT mice (Figure 4A,B). The time spent by TAAR5-KO mice in the open arms was significantly shorter after the predator exposure in comparison with that measured before the predator stress (249 ± 25 s and 144 ± 22 s, respectively; *p* < 0.05, paired two-tailed *t*-test). The same parameter measured in WT mice was not different between pre- and post-stress values (158 ± 23 s and 168 ± 22 s, respectively, paired two-tailed *t*-test). The preference to stay in closed arms following the stress was found for both genotypes (190 ± 25 s vs. 232 ± 25 s for WT mice and 104 ± 34 vs. 221 ± 14 s for TAAR5-KO mice, *p* < 0.05, paired two-tailed *t*-test).

The predator stress exposure differently altered cognitive performance in WT and TAAR5 mice, which was assessed in the NOR test (Figure 4C,D). Thus, the total exploration time (both new and old objects) was not different between pre- and post-stress values in WT animals (37 ± 12 s vs. 21 ± 8 s, respectively; *p* = 0.46, paired two-tailed *t*-test). However, the same measure was significantly lower in TAAR5-KO mice after the stress versus before stress value (6 ± 2 s and 31 ± 6 s, respectively; *p* < 0.05, paired two-tailed *t*-test). On the contrary, the discrimination index was significantly decreased after the stress in WT control (0.5 *±* 0.1 before vs. −0.9 *±* 0.2 after; *p* < 0.05, paired two-tailed *t*-test) and surprisingly was not different in mutants (0.2 ± 0.1 and −0.1 *±* 0.3; *p* > 0.05, paired two-tailed *t*-test).

Finally, the predator stress had no influence on sucrose and water consumption in both groups (Figure 4E,F). Sucrose consumption pre- and post-stress values in WT (40.6 ± 2.0 g vs. 44.7 ± 7.6 g, respectively; *p* > 0.05, paired two-tailed *t*-test) and in TAAR5-KO mice (50.4 ± 5.9 g vs. 37.7 ± 3.7 g, respectively; *p* > 0.05, paired two-tailed *t*-test). Water consumption in WT (2.7 ± 0.3 g vs. 2.8 ± 0.1 g, respectively; *p* > 0.05, paired two-tailed *t*-test) and in TAAR5-KO mice (2.7 ± 0.4 g vs. 2.8 ± 0.2 g, respectively; *p* > 0.05, paired two-tailed *t*-test). Sucrose preference for water in WT (93.7 ± 0.6% before vs. 94.0 ± 0.4% after; *p* > 0.05, paired two-tailed *t*-test) and in TAAR5-KO mice (94.3 ± 1.0% before vs. 92.5 ± 1.3% after; *p* > 0.05, paired two-tailed *t*-test). A concise summary on all behavioral and neurochemical parameters, which mice exhibited throughout this study, is presented in Table 1.

## 4. Discussion

FSCV experiments showed that electrically evoked DA release is significantly increased in the NAc of TAAR5-KO mice compared to their WT counterparts. However, no difference in DA uptake was found between these genotypes. Behavioral evaluation confirmed a reduced anxiety level measured as time spent in both closed and open arms of EPM in mutant mice. The NOR test did not uncover the difference between TAAR5-lacking and WT mice. In the sucrose drinking paradigm, TAAR5-KO mice consumed more sugar solution than the control group, while water intake was not different. During a predator stress exposure, mutant and WT mice displayed quite distinct responses. Expectedly, the control group demonstrated a substantial increase in “freezing”, while TAAR5-KO mice showed no significant changes versus analogous behavior observed under stressless conditions. In contrast to the “freezing” pattern, “running” and “exploring” were significantly increased in mice lacking TAAR5, but not in controls (some trend toward significance was evident for “freezing” in WT mice). “Grooming” was not altered in either genotype during exposure to the predator. Short-term consequences of stress were explored 24 h after exposure to a predator in the EPM, NOR, and sucrose drinking tests. In the EPM test, intact TAAR-KO mice showed less anxiety—related behaviors as shown previously [1], but following stress exposure, mutant mice displayed heightened levels of anxiety in both open and closed arms, while WT control mice showed an increase just in closed arms. Noticeably, the stress resulted in spending equal time in closed arms by both groups, whereas the basal anxiety was significantly lower in mutants. In the NOR test, the predator exposure decreased total exploration time in TAAR5-KO and WT mice to the same extent. Surprisingly, following the interaction with the predator, mutants distinguished a new object without changes (similarly to the pre-stress condition), while WT mice displayed a significantly decreased discrimination index. As a matter of fact, the predator stress did not influence animals’ hedonic function: sucrose consumption under pre- and post-stress assessment was not affected in both genotypes.

Previous studies revealed that TAAR5-KO mice have a distinct behavioral phenotype, which is characterized by reduced anxiety-like behaviors [1] and better cognitive performance in the decision-making task due to a reduced number of errors, and displayed a greater rate of improvement [14]. The current work further confirmed the anxiolytic phenotype of these mice by the findings that mutants spend more time in open arms and less time in closed arms of EPM in comparison with WT controls. Anxious conditions are tightly connected to the function of the brain serotonin transmission [38]. Indeed, the measurements of tissue serotonin content in several brain regions revealed its reduced level in the striatum and hippocampus, while the main metabolite of serotonin, 5-HIAA, was decreased in the hippocampus and hypothalamus of TAAR5-KO mice [1]. Moreover, the selective serotonin 1A receptor agonist (8-OH-DPAT) induced enhanced hypothermic response in mutants [1]. Altogether, these results may offer a plausible mechanistic explanation for the anxiolytic-like phenotype of mice with TAAR5 deletion [1].

However, the effect of TAAR5 deletion on other monoamine systems, which are tightly involved in emotional behaviors, should be taken into account for the phenotype. Indeed, we found that DA release is markedly increased in the brain region, where this neurotransmitter is implicated in the control of emotions, motivation, and learning, and therefore might dramatically shape the behavior of mutants. Remarkably, a previous optogenetic study discovered decreased anxiety resulting from NAc stimulation. Specifically, stimulated mice exhibited more entries into the open arms of the EPM [39]. Furthermore, accumbal DA opto-activation increased appetitive behavior [39] that aligns with the augmented sugar intake and enhanced DA release in TAAR-KO mice.

Our voltammetry data are in agreement with earlier findings that mice lacking TAAR5 have an increased number of dopaminergic neurons and subsequently increased DA and its metabolites tissue content, as well as DA and neurogenesis-related signaling events [2,4]. These changes are more likely responsible for the enhanced terminal DA efflux observed in our experiments after the stimulation of DA cell bodies. The enhanced rate of DA reuptake due to an upregulation in DA transporter is a common presynaptic compensatory response to the increased extracellular dopamine [20,40]. However, the analysis of DA dynamics did not reveal a significant difference in the maximal reuptake rate (V max) between mutants and WT animals. Further studies are warranted to determine whether the DA synthesis rate and its autoreceptor function are secondarily involved in the effect of TAAR5 deletion. Therefore, genetic elimination of the receptor leads to alterations in central monoaminergic neurotransmissions, specifically serotonergic and dopaminergic, the equilibrium of which is crucial for sustaining certain emotional conditions, aiming to efficiently adjust to environmental changes [7].

Stress is one of the most powerful challenges that triggers two opposite types of stress-coping strategies, passive and active, which allow an organism to deal with perceived threats. The subject interaction with the predator (resident rat) in the resident-intruder paradigm is extremely stressful and capable of inducing PTSD-like consequences [9,10,11,12]. Therefore, the shift of behavior in WT mice to the escalated “freezing”, which is the preferential pattern of passive coping, perhaps, should not be surprising under this condition. In sharp contrast to the control, TAAR5-KO mice did not display a significant increase in this behavioral manifestation, whereas “running” and general activity as “exploring” were markedly enhanced. These observations indicate that active coping may be the preferred strategy for mutant mice to deal with predator stress. Perhaps an increased dopaminergic activity (concurrent with decreased serotonergic tone) induced by genetic TAAR5 removal could result in this dissimilar behavior. Fascinatingly, triggering DA release in the mouse NAc during social defeat through optogenetic stimulation reorganized behavior towards resilience-associated patterns [39]. These findings provide a causal link between accumbal DA transmission and behavioral strategy under stress conditions. Nevertheless, there are other downstream targets of the VTA dopaminergic neurons, including the medial prefrontal cortex and basolateral amygdala (BLA), which could contribute to fear-triggered responses [41,42]. In fact, TAAR5 expression was also detected in these brain regions [1]. A recent fiber photometry study found that DA activity in the BLA fear extinction neurons is time-locked to freezing cessation [42]. It is possible that the hyperdopaminergic state in TAAR5-KO mice is not restricted to accumbal terminals but extends to other brain areas, including the BLA. If it is the case, an increased DA release in this region could at least partly explain the suppressed “freezing” pattern in mutants during predator stress. However, future neurochemical studies are needed to evaluate the status of the BLA DA system in the absence of TAAR5.

It should be highlighted that TAAR5 was highly expressed in the olfactory epithelium. A second class of chemosensory receptors in the olfactory epithelium [43], a crucial domain for the perception of odors and the sending of this information to other parts of the brain for the interpretation and consequent action. At the same time, TAAR5 is also located in the limbic brain areas processing olfactory information [1]. The predator odor detection should help the mouse to initiate an appropriate fear response to a specific threat. It is possible that TAAR5 mutants placed in the cage with a predator cannot effectively identify critical salient signals from a dangerous environment, since the brain obtains insufficient information. Therefore, their behavior during acute stress would be shaped quite differently from WT mice due to the sensory confusion.

A strong acute stressor might result in long-term psychopathological syndromes such as PTSD. However, behavioral manifestations, indicating signs of anxiety and depression, can also be evident on a short-term scale. Thus, depressive-like alterations were developed in rats within 24 h after a single social defeat experience with an aggressive opponent [17,18,44,45]. Here, short-term predator stress consequences were explored in mice. Based on the findings of lessened basal anxiety and more fearless responses during the interaction with the predator, it could be expected that TAAR5-lacking mice following stress should display reduced anxiety. However, the absence of TAAR5 did not protect mice from the development of anxious behavior. The level of anxiety in mutants reached that observed in control mice. Moreover, total exploration time in the NOR test was decreased in mutants, and these changes are more likely due to stress-induced anxiety. This is in agreement with potential dysfunction of the stress-response circuitries in the brain. The sensory deprivation in TAAR5-KO mice may be a key factor for this consequence. The lack of a direct threat identification can be severely stressful itself and, therefore, results in profound anxiety after a subject is disconnected from a fearful environment.

Fascinatingly, mutant mice continued to discriminate between new and old objects, while WT mice could not do it after the stress exposure. In addition to their anxiolytic- and antidepressant-like properties [1], TAAR5-KO mice display improved cognitive performance in some tasks [14]. Therefore, the ability of mutant mice to distinguish objects under escalated anxious conditions suggests that cognitive domains can be more resistant to acute stress than emotional processes.

## 5. Conclusions

This study provides compelling evidence that TAAR5 is an important player in the modulation of the mesolimbic dopaminergic system and in processing stress-coping strategies and short-term emotional outcomes. Our findings demonstrate that genetic deletion of TAAR5 results in a hyperdopaminergic state within the NAc. This neurochemical alteration is associated with a distinct behavioral phenotype, including reduced baseline anxiety and increased sucrose preference. Interestingly, while TAAR5-KO mice exhibit a more active coping strategy during acute predator stress, this does not confer full stress resilience. On the contrary, these mutants developed after predator stress somewhat more anxiety-like behaviors, unlike their wild-type counterparts.

Therefore, we propose a dual role for TAAR5: its presence is necessary to maintain tonic inhibitory control over mesolimbic dopamine pathways, and its activity is crucial for orchestrating an appropriate and adaptive stress response that prevents maladaptive psychopathological outcomes. Future research should focus on exploring TAAR5 as a promising novel therapeutic target for conditions such as PTSD and anxiety disorders.

## Figures and Tables

**Table 1:** The summary table illustrates behaviors of TAAR5-KO and WT mice before, during, and after the predator exposure.

Conditions	Groups	
	TAAR5-KO	WT
No stress	↓ Anxiety ↑ Mesolimbic dopamine ↑ Sucrose consumption	Normal behavior
During the predator exposure	Active behavior	Passive behavior
24 h after the predator exposure	↑↑ Anxiety, ↓ exploration	↑ Anxiety, ↓ cognition

The arrows facing up demonstrate an enhancement in the indicated parameter but those facing down-a reduction.

## Data Availability

The data presented in this study are available from the corresponding author upon reasonable request.
